# Changes in the Biophysical Properties of the Cell Membrane Are Involved in the Response of *Neurospora crassa* to Staurosporine

**DOI:** 10.3389/fphys.2018.01375

**Published:** 2018-10-11

**Authors:** Filipa C. Santos, Gerson M. Lobo, Andreia S. Fernandes, Arnaldo Videira, Rodrigo F. M. de Almeida

**Affiliations:** ^1^Departamento de Química e Bioquímica, Faculdade de Ciências, Centro de Química e Bioquímica, Universidade de Lisbon, Campo Grande, Lisbon, Portugal; ^2^I3S - Instituto de Investigação e Inovação em Saúde, Universidade do Porto, Porto, Portugal; ^3^IBMC-Instituto de Biologia Molecular e Celular, Universidade do Porto, Porto, Portugal; ^4^ICBAS-Instituto de Ciências Biomédicas de Abel Salazar, Universidade do Porto, Porto, Portugal

**Keywords:** antifungal drug, plasma membrane, sphingolipid domains, biophysical properties, ergosterol, liposomes, fluorescence spectroscopy, conidial development

## Abstract

*Neurospora crassa* is a non-pathogenic filamentous fungus widely used as a multicellular eukaryotic model. Recently, the biophysical properties of the plasma membrane of *N. crassa* conidia were thoroughly characterized. They evolve during conidial germination at a speed that depends on culture conditions, suggesting an important association between membrane remodeling and the intense membrane biogenesis that takes place during the germinative process. Staurosporine (STS) is a drug used to induce programmed cell death in various organisms. In *N. crassa*, STS up-regulates the expression of the ABC transporter ABC-3, which localizes at the plasma membrane and pumps STS out. To understand the role of plasma membrane biophysical properties in the fungal drug response, *N. crassa* was subjected to STS treatment during early and late conidial development stages. Following 1 h treatment with STS, there is an increase in the abundance of the more ordered, sphingolipid-enriched, domains in the plasma membrane of conidia. This leads to higher fluidity in other membrane regions. The global order of the membrane remains thus practically unchanged. Significant changes in sphingolipid-enriched domains were also observed after 15 min challenge with STS, but they were essentially opposite to those verified for the 1 h treatment, suggesting different types of drug responses. STS effects on membrane properties that are more dependent on ergosterol levels also depend on the developmental stage. There were no alterations on 2 h-grown cells, clearly contrasting to what happens at longer growth times. In this case, the differences were more marked for longer STS treatment, and rationalized considering that the drug prevents the increase in the ergosterol/glycerophospholipid ratio that normally takes place at the late conidial stage/transition to the mycelial stage. This could be perceived as a drug-induced development arrest after 5 h growth, involving ergosterol, and pointing to a role of lipid rafts possibly related with an up-regulated expression of the ABC-3 transporter. Overall, our results suggest the involvement of membrane ordered domains in the response mechanisms to STS in *N. crassa*.

## Introduction

Antifungal drug resistance is a major society concern, since the mortality rate associated with resistant fungal infections has increased dramatically, particularly in hospital environments and in patients with decreased immunological response (Gulshan and Moye-Rowley, 2007; Shahi and Moye-Rowley, 2009; Prevention CFDCA., 2017).

The emerging role of apoptosis as key regulator of fungal development suggests that it might be possible to develop new means of controlling fungal infections through the manipulation of some key components/organelles involved in the apoptotic cascade. In fungi, apoptotic-like cell death occurs naturally during developmental processes and reproduction, and can be induced by environmental factors and exposure to toxic metabolites or abiotic factors. The core apoptotic processes in fungi are similar to those in mammals, however, the apoptotic network is less complex (Sharon et al., 2009).

The alkaloid staurosporine (STS) is well-known for its antifungal (Omura et al., 1977; Park et al., 2006) and antitumoral characteristics (Correa et al., 2011), but also for being the most potent protein kinase inhibitor, with a half maximal inhibitory concentration *in vitro* in the nanomolar range (Tamaoki et al., 1986), and an inducer of programmed cell death in neuronal cells (e.g., Wiesner and Dawson, 1996), protozoans (e.g., Yin et al., 2010) human macrophages (e.g., Dunai et al., 2012) and in the filamentous fungus *Neurospora crassa* (Gescher, 2000; Castro et al., 2010; Fernandes et al., 2011, 2013). STS-induced programmed cell death in filamentous fungus (Fernandes et al., 2011) is thus one of the many examples of important fungal physiological activities, such as cell proliferation or differentiation, sensing and signaling, that are usually found to be closely related to membrane composition and biophysical features, through their involvement with membrane microdomain organization and lipid homeostasis (Malinsky and Operakova, 2016). Indeed, a gene expression (microarray) study (Fernandes et al., 2011) shows that there are important changes in the levels of mRNA coding for several enzymes of lipid metabolism and also of (signaling) proteins that interact with the membrane and may affect domain formation and properties (Table S1). The plasma membrane protein for which mRNA levels determined in the transcriptional profiling study are increased by a larger amount, attaining a 30-fold increase is ABC- 3 (Fernandes et al., 2011). Moreover, this protein is responsible for most of the energy-dependent efflux of STS and a null-mutant of this ABC transporter (abc3) is extremely sensitive to STS and accumulates more STS than the wild type strain (Fernandes et al., 2011).

In the present study, we use the biological model *N. crassa* conidial cells and STS to biochemically characterize fungal plasma membranes when challenged with an antifungal drug. We asked whether there could be a biophysical response at the plasma membrane level that could be consequence of STS challenge. This drug does not directly target the cellular envelope or any protein activity involved in membrane lipid synthesis and catabolism, contrary to many antifungal drugs, such as polyenes, azoles, and sphingoid base analogs, but might significantly change the plasma membrane composition, as indicated by transcriptomic analysis. The use of STS will disclose if even in such a case membrane lipid composition and biophysical properties should be considered to understand the physiological response of filamentous fungi to the drug. Until recently, the biophysical properties of conidial cell membrane, namely of *N. crassa*, were practically unknown, but a thorough biophysical characterization of *N. crassa* plasma membrane was carried out and important biophysical properties of the plasma membrane of *N. crassa* conidia are now well characterized, as well as their dynamic behavior along conidial germination (Santos et al., 2017). The cell membrane becomes globally more fluid with growth time, it contains ordered sphingolipid-enriched domains that differ from the ones known as lipid rafts since they have no ergosterol, and the gel-like nature of those domains, despite being much less rigid than those found in the yeast *Saccharomyces cerevisiae* (Aresta-Branco et al., 2011), leads to a higher global membrane order than in the budding yeast (Santos et al., 2017).

In the present work, we studied the biophysical effects of STS on the plasma membrane lipid domains of *N. crassa* and also evaluated if the drug interacts with lipids of the plasma membrane. We show, in fact, that the mechanism of STS response involves changes in membrane biophysical properties dependent on lipid organization and composition, and thus these should be taken into account when studying the mechanism of STS action and the physiological responses of filamentous fungi to drugs. Using this drug also provides an opportunity to disclose if there could be a fast response involving reorganization of lipids and lipid domains at the plasma membrane before the genetic response has fully emerged adding to knowledge on antifungal action and providing clues to a better understanding of antifungal drug response by filamentous fungi.

## Materials and methods

### Strains, growth techniques and chemicals

*N. crassa* wild type strain (FGSC 2489) and the null-mutant of the ABC transporter ABC-3 (abc3) (FGSC 14572) were obtained from the Fungal Genetics Stock Center (Mccluskey, 2003). Standard procedures were employed for growth and handling of *N. crassa* in Vogel's Minimal Medium (Davis et al., 1970).

STS was obtained from LC-Laboratories, DPH (1,6-diphenyl-1,3,5-hexatriene) and di-4-ANEPPS (4-(2-(6-(dibutylamino)-2-naphthalenyl)ethenyl)-1-(3-sulfopropyl)-pyridinium) were obtained from Invitrogen (Madrid, Spain). The probe *t*-PnA (*trans*-parinaric acid) was purchased from Santa Cruz Biotech. (Santa Cruz, C.A.). Ludox® (colloidal silica diluted to 50% in water) was purchased from Sigma-Aldrich (St. Louis, MO). Solvents/co-solvents such as ethanol, methanol, and glycerol were spectroscopic grade and purchased from Merck and Scharlau. All other reagents were of the highest purity available. Stock solutions of STS (13.2 mM STS in DMSO) and of fluorescent probes were quantified spectrophotometrically (de Almeida et al., 2005; Bastos et al., 2012a). For fluorescence spectroscopy studies, STS was diluted in PBS at 700 μM before use.

All the results obtained are presented as the mean ± standard deviation (S.D.) obtained from independent liposomal suspensions/ biological replicates, and statistical significance was determined using Student's *t*-test. Mean values were considered significantly different for *p* values below 0.05.

### Fluorescence spectroscopy of STS in liposomes

Liposome (multilamellar vesicles) suspensions of 3 mM lipid were used. This high lipid concentration was used to ensure that even a weak interaction with the membrane could be perceived. Three different lipid compositions were prepared: DPPC (1,2-dipalmitoyl-*sn*-glycero-3-phosphocholine), POPC (1-palmitoyl-2-oleoyl-*sn*-glycero-3-phosphocholine), and a binary mixture of DPPC/ Cholesterol (1:1 mol:mol). Cholesterol was chosen instead of ergosterol because ergosterol absorbs light in the same range as STS and, as it corresponds to 50 mol% of the lipid, is present in a much higher concentration (1.5 mM vs. 12.5 μM), precluding reliable measurements of STS fluorescence.

To prepare the liposome suspensions, lipid stock solutions were added to a glass tube and the solvent was slowly vaporized by a mild flow of nitrogen, forming a thin layer of lipid. The lipid was hydrated by the addition of 1 mL of PBS previously heated above the main transition temperature (*T*_m_) of the lipids. The samples were then progressively vortex-stirred and submitted to at least 5 freeze/thaw cycles. Afterwards, STS was added to the prepared multilamellar vesicles suspensions to a final concentration of 12.5 μM, and the suspension was incubated at room temperature (23 ± 2°C). After 1 h, the liposome suspensions were analyzed by steady-state and time-resolved fluorescence spectroscopy, taking advantage of the intrinsic fluorescence of the drug, using excitation and emission wavelengths of 290 and 377 nm, respectively, and bandwidth of 1.5 nm in spectral acquisition and 3 nm in anisotropy measurements. STS in PBS was used as control. Liposome suspensions without STS were used as blanks. Both steady-state and time-resolved fluorescence measurements were performed with a Horiba Jobin-Yvon Spex Fluorolog 3.22, at 30°C in a temperature-controlled sample compartment under magnetic stirring. For time-resolved measurements by the single photon counting technique, a nanoLED N-280 for excitation at 279 nm; emission was collected at 377 nm with a bandwidth between 5 and 7 nm. The other experimental conditions and data analysis follow the procedure described below for the studies with cell suspensions.

### STS challenge and fluorescence spectroscopy in cells

*N. crassa* conidia were grown in liquid minimal medium at a starting concentration of 10^7^ cell/mL, at 30°C and 150 rpm. After 2 or 5 h of growth, each culture was submitted to a 12.5 μM STS challenge and cells were further incubated for 1 h. In parallel, 3 or 6 h grown cultures were challenged with 12.5 μM STS during 15 min, under the same conditions. The same volume of PBS was added to control cultures. This 1 h delay ensured that the total growth time of the controls was similar in both sets of experiments, i.e., 3 and 6 h or 3 h 15 min and 6 h 15 min, respectively.

Cells were washed by centrifugation at 10,000 *g* for 2 min and resuspended in PBS.

The fluorescence spectroscopy procedures used in the present work were previously optimized (Santos et al., 2017). As before, the fluorescent probes *t*-PnA (2 μM), DPH (2 μM), and di-4-ANEPPS (1 μM) were added to the cells and incubated for 10 min at 30°C unless otherwise stated. For steady-state anisotropy measurements, excitation and emission wavelengths of 320 and 404 nm (*t*-PnA) or 360 and 425 nm (DPH) were used. Bandwidths of 4 nm were used for both *t*-PnA and DPH. For di-4-ANEPPS spectra acquisition, bandwidths of 4 nm were used.

The membrane dipole potential was measured through the ratio of excitation intensities at 420 and 520 nm of di-4-ANEPPS with emission at 635 nm. The *R*_*ex*_ can be linearly related with the membrane dipole potential under the conditions met in this study [30–33], hence the excitation spectra were acquired with emission wavelength of 635 nm, which is at the red edge of the spectrum, allowing to rule out the membrane fluidity effects, and as referred above there were no shifts observed in the spectra upon the STS challenge. Thus, the dipole potential (ψ_d_) in mV was calculated from *R*_*ex*_ using the linear relationship (Equation 1) (Haldar et al., 2012):

(1) $$\begin{array}{l} \psi_{d}=\frac{R_{ex}+0.3}{4.3\times 10^{-3}} \end{array}$$

The steady-state fluorescence anisotropy (*r*) was calculated according to,

(2) $$\begin{array}{l} r=\frac{\left(I_{VV}-G\times I_{VH}\right)}{\left(I_{VV}+2G\times I_{VH}\right)} \end{array}$$

in which *G* is the instrumental correction factor and the subscripts *V* and *H* represent the vertical and horizontal orientations of the polarizers. The order of the subscripts corresponds to excitation and emission. An adequate blank was subtracted from each intensity reading.

For time-resolved measurements by the single photon counting technique, a nanoLED N-320 and a nanoLED N-460 were used for excitation of *t*-PnA and di-4-ANEPPS, respectively. Emission was set to 404 and 634 nm, respectively. Ludox® was used as the scatterer to obtain the instrumental response function. The program TRFA data processor version 1.4 (Minsk, Belarus) was used for the analysis of the experimental fluorescence decays. To describe the decays, a sum of exponentials with α_i_ the normalized amplitude and τ_i_ the lifetime of component *i*, was used:

(3) $$I\left(t\right)=\sum_{i=1}^{n}\alpha_{i}\text{ exp }\left(-\frac{t}{\tau_{i}}\right)$$

The amplitude-weighted mean fluorescence lifetime was calculated as follows,

(4) $$\tau_{av}=\sum_{i=1}^{n}\alpha_{i}\tau_{i}$$

and the intensity-weighted mean fluorescence lifetime was obtained through the following expression,

(5) $$<\tau >=\frac{\sum_{i=1}^{n}\alpha_{i}\tau_{i}^{2}}{\sum_{i=1}^{n}\alpha_{i}\tau_{i}}$$

The quality of the fit was judged by random distribution of weighted residuals and residuals autocorrelation and a reduced χ^2^ value close to 1.

### SDS-page and western blot

*N. crassa* at a starting concentration of 10^7^ conidia/mL was grown in 10 mL liquid minimal medium at 30°C and 150 rpm, followed by the addition of 12.5 μM STS or same volume of DMSO, and incubated under the same conditions. Cells were collected by fast filtration. Total protein extracts were obtained from disruption with zirconia beads in a FastPrep-24 (MP Biomedicals). SDS-PAGE and western blot using an antibody against ABC-3 protein were performed as described in Fernandes et al. (2011).

## Results

### STS-lipid interactions

To study a possible interaction of STS with membrane lipids, three different model systems were used, covering the three lipid bilayer phases thought to represent the most important types of lipid domains in fungal plasma membrane (Rosetti et al., 2017): gel [composed of DPPC, with a *T*_m_ value 41.5°C (Huang and Li, 1999)], liquid disordered [composed of POPC, *T*_m_ ca. −3°C (Koynova and Caffrey, 1998)], and liquid ordered [composed of a binary mixture of DPPC/cholesterol, which is liquid ordered at both room temperature and 30°C (de Almeida et al., 2007)]. Moreover, due to STS intrinsic fluorescence, this study could be performed without resorting to externally added labels.

Different photophysical parameters of STS were measured. The steady-state fluorescence anisotropy reflects the rotational mobility of the drug, which should be significantly restricted upon membrane adsorption or incorporation. The fluorescence intensity decay allows computing the amplitude-weighted and intensity-weighted mean fluorescence lifetime, after analysis using equation 3 with a bi-exponential model (*n* = 2) and equations 4 and 5. Those two parameters reflect the microenvironment of the probe: the contribution of each of them to the total decay can change with the solvent polarity and specific interactions, or with the pathways the excited fluorophore follows to return to the ground state, which depend on collisions with other molecules, vibrations and torsions (Berezin and Achilefu, 2010).

**Figure 1:**
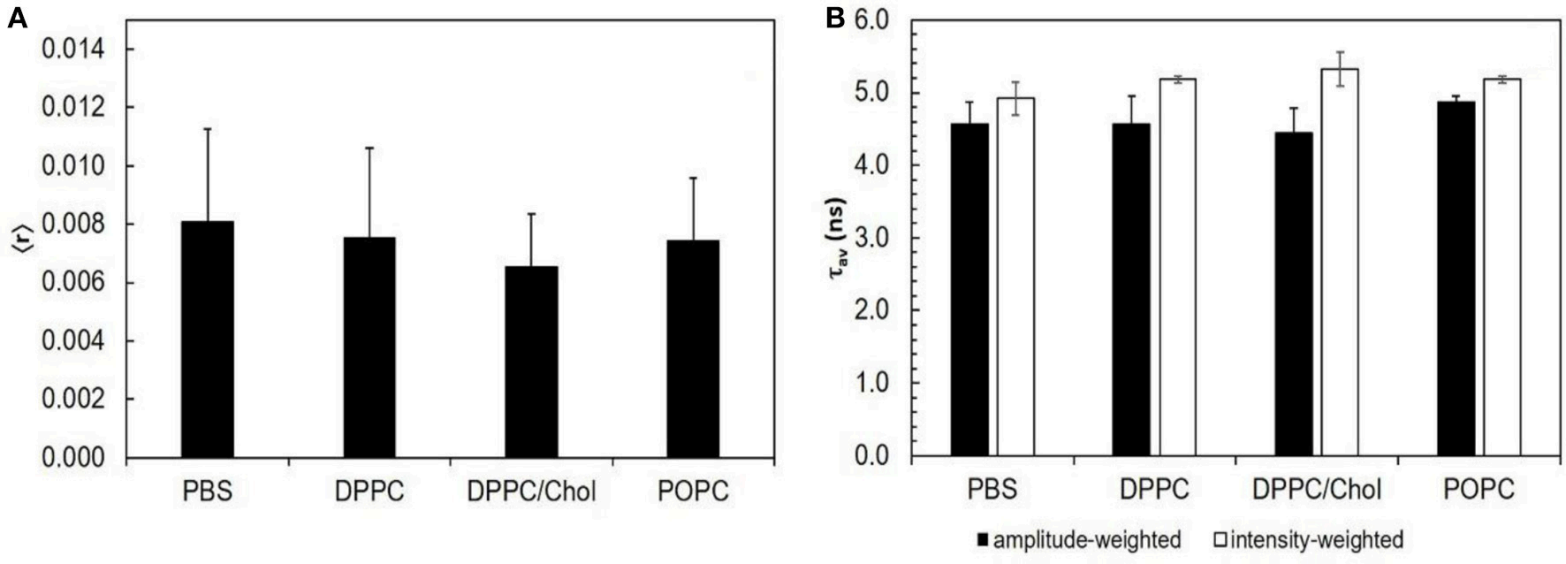
STS does not interact with lipid bilayers: the intrinsic fluorescence properties of STS are similar in PBS and in the presence of different lipid phases. **(A)** steady-state fluorescence anisotropy; **(B)** amplitude-weighted (black bars) and intensity-weighted (white bars) mean fluorescence lifetime. The results represent the mean ± S. D. of at least three independent experiments, *n* ≥ 3.

None of the STS parameters analyzed were significantly altered by the presence of any of the model systems used (Figure 1). These results show that STS does not interact directly with the membrane lipids. The variations detected in the conidial plasma membrane (next section) must therefore arise from active membrane reorganization and/ or lipid composition alterations actively performed by the cell.

### ABC-3 expression at the plasma membrane: influence of germination and STS exposure time

The expression of membrane proteins, such as the ones from the ATP Binding Cassette (ABC) transporter family, is an important part of antifungal drug response. These are primary active transporters involved in the modulation of absorption, metabolism and toxicity of pharmaceutical drugs (Glavinas et al., 2004). From this family, the ABC-3 transporter of *N. crassa*, homologous to the human P-glycoprotein 1 (Cannon et al., 2009), is responsible for the STS efflux (Fernandes et al., 2011). As mentioned above, this is the single protein whose levels of mRNA showed by far, for specific conditions, the largest increase (Fernandes et al., 2011). Therefore, we chose to study in more detail the expression of this protein at the plasma membrane because it is expected to observe very clear trends that can be used in combination with our previous biophysical study along conidial germination to choose a feasible number of experimental conditions that clearly complement each other as far as STS physiological response of *N. crasssa* conidia is concerned.

**Figure 2:**
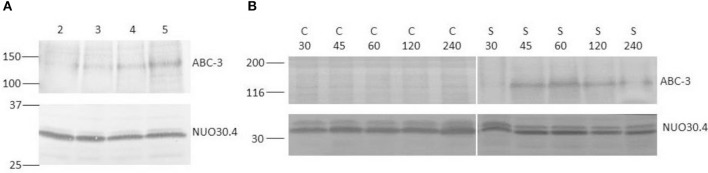
Western blots of total protein extracts from *N. crassa* cells, using antiserum against the 130 kDa ABC-3 and the constitutive 30.4 kDa subunit of complex I (NUO30.4), as control for loading. **(A)** *N. crassa* was grown for the indicated times in hour and subsequently exposed to 12.5 μM STS for 1 h. **(B)** *N. crassa* was grown for 5 h and subsequently incubated with 12.5 μM STS (S) or DMSO as control (C) for the indicated times in min. Standard molecular weights (kDa) are indicated on the left of each blot.

In Figure 2, the Western blots for ABC-3 are shown for different growth times and different STS time exposure. However, we used only one concentration of STS. Previous studies from Castro et al. (2010), show that the concentration and incubation times used here ensure high survival rates and noticeable physiological effects. Hence, the concentration of STS used in the previous work by Fernandes et al. (2011) for most of the experiments was already 12.5 μM. Moreover, there were practical reasons to choose this concentration. Lower values of STS would yield low absorbance or fluorescence intensity values, which would introduce larger uncertainty in the study of STS intracellular accumulation (Fernandes et al., 2011). Larger concentration values could interfere with the fluorescence studies using *t*-PnA, since there is a large overlap between the absorption and emission spectra of this membrane probe and those of STS. As detailed below, *t*-PnA is an essential probe in membrane biophysical studies because of its unique sensitivity to acyl chain packing in the most ordered lipid domains, i.e., to study the important sphingolipid-enriched plasma membrane domains.

STS at a concentration of 12.5 μM induces ABC-3 expression after 1 h of incubation but much more markedly in 5 h-grown cells than in 2 or 3 h-grown cells (Figure 2A), and the expression is only detected after 45 min (Figure 2B), which means that after 15 min exposure there is practically no overexpression at the plasma membrane.

### Biophysical changes of plasma membrane lipids upon STS challenge

To assess the influence of germination time and duration of STS stimulus, comparison will be attempted for the four combinations of 2 and 5 h growth plus 1 h drug challenge and 3 and 6 h plus 15 min drug challenge, and respective controls. To facilitate the analysis of the results, they are presented as *N. crassa* cells in the absence (full pattern) and in the presence of STS (stripped pattern) with incubation time of 1 h (black colored) and of 15 min (gray colored). These two growth times allow comparison of the fungal drug responses at the early vs. late conidial stages of development, ensuring at the same time complementary information regarding the expression of ABC-3 at the plasma membrane. The STS treatment times were chosen taking into account the data presented in Figure 2B, where it is possible to observe the highest levels of protein ABC-3 expression between the STS incubation times of 45 min and 60 min, thus we have chosen the STS 1 h treatment. For a more complete study we decided to perform a 15 min treatment with STS, to ensure that the increase of ABC-3 expression levels at the plasma membrane were still undetectable, even for a growth time of 5 h. This allows assessing if we can distinguish a fast vs. a slow response to the drug in terms of membrane biophysical properties in conditions where the expression of large membrane proteins such as ABC transporters are unchanged or not.

#### Packing and order of the acyl chains

The fluorescence intensity decay of *t*-PnA can be used to identify the presence of ordered domains. These can be attributed to a gel phase in the presence of a very long lifetime component in the fluorescence decay (Aresta-Branco et al., 2011; Bastos et al., 2012b; Vecer et al., 2014) or to a liquid ordered phase (de Almeida et al., 2009). In *N. crassa* conidia, the very low levels of ergosterol impede the formation of detectable amounts of liquid ordered domains by *t*-PnA (Santos et al., 2017). The lifetime value of the long component is related to the packing efficiency of the acyl chains. In turn, the amplitude of the long component is related to the relative abundance of those ordered domains.

**Figure 3:**
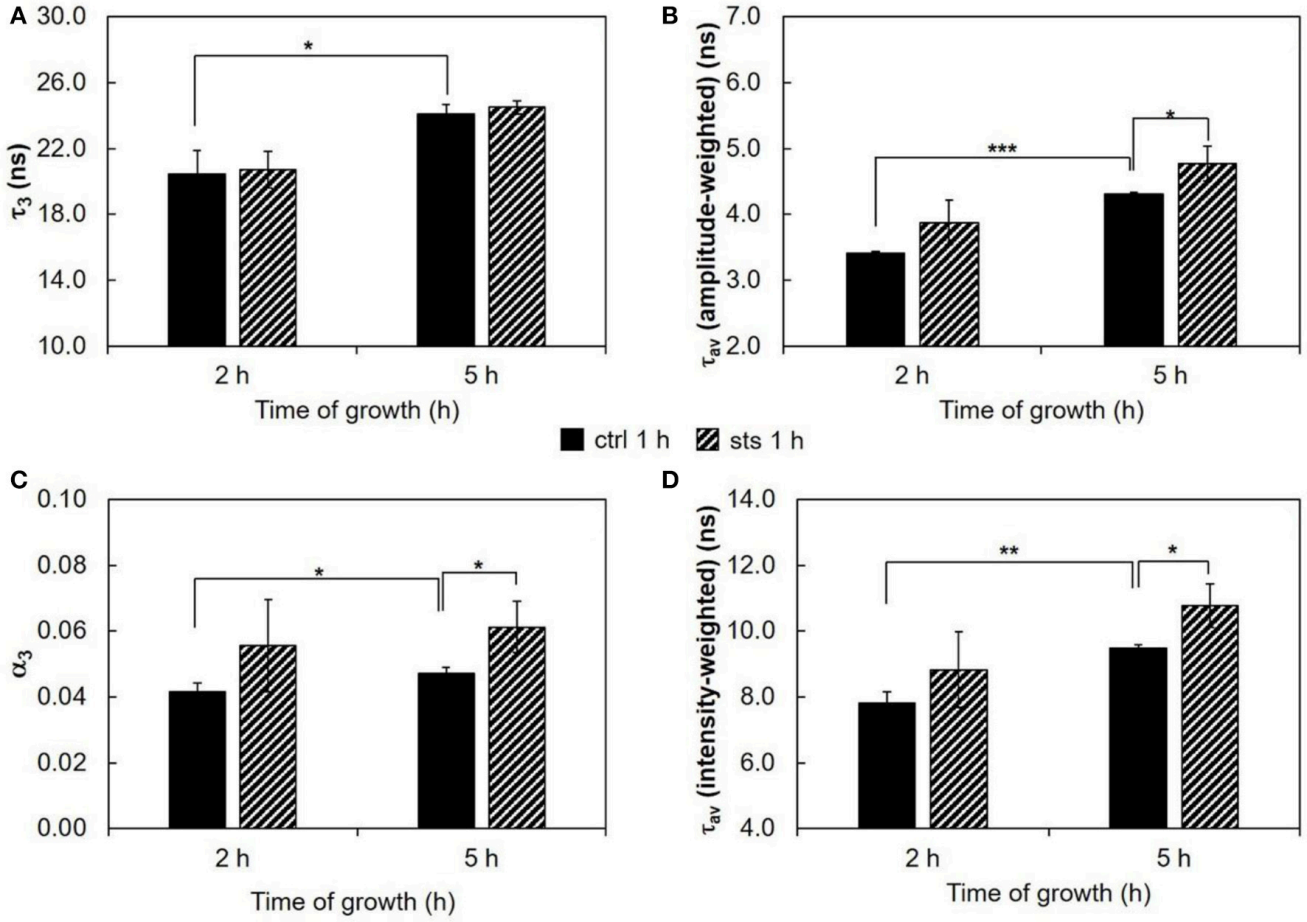
Time-resolved fluorescence spectroscopic parameters of *t*-PnA in the plasma membrane of *N. crassa* conidia. **(A)** The long lifetime component, τ_3_, **(B)** amplitude-weighted mean fluorescence lifetime, τ_av_ (amplitude-weighted), **(C)** normalized amplitude of the long component, α_3_, and **(D)** intensity-weighted mean fluorescence lifetime, τ_av_ (intensity-weighted), of *t*-PnA were obtained from the fluorescence intensity decay of the probe at 30°C. The results present the mean ± S.D. of at least three independent experiments, *n* ≥ 3. ^*^p < 0.05, ^**^p < 0.01, ^***^p < 0.001.

Commencing with the 1 h challenge, STS did not have a major impact on the long lifetime component of *t*-PnA (Figure 3A and Table S2). This parameter increased from ~20 ns at 2 h to ~25 ns at 5 h growth, and was independent of the presence of STS, reflecting the presence of ordered domains as reported previously by us (Santos et al., 2017). These are sphingolipid-enriched domains that are not highly rigid gel, because their melting temperature is very close to *N. crassa* growth temperature. However, significant effects were observed for the relative abundance of the sphingolipid-enriched domains, since the amplitude associated with the long lifetime component increased in the presence of STS at 5 h of growth (Figure 3C). Regarding the mean fluorescence lifetimes of *t*-PnA, the general trend is to observe an increase of their values induced by STS (Figures 3B,D).

**Figure 4:**
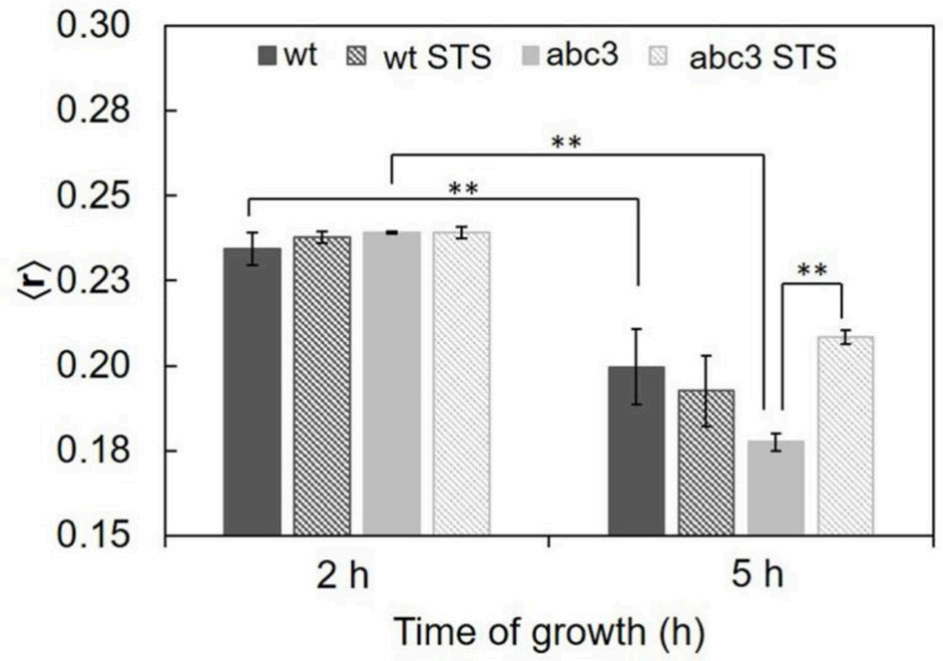
Steady-state fluorescence anisotropy of DPH in the plasma membrane of *N. crassa* conidia, at 30°C of the wild type (wt) or abc3 mutant, after 1 h incubation in the absence or presence of 12.5 μM STS. The values are the mean ± S.D. of at least three independent experiments, *n* ≥ 3. ^**^p < 0.01.

The ordering or disordering effects of STS in the membrane were also assessed. The steady-state fluorescence anisotropy values of DPH in the controls were similar to previously reported (Santos et al., 2017). DPH, not being sensitive to any particular kind of domain, gives a view of the plasma membrane global order. The values of steady-state fluorescence anisotropy of DPH decrease from 2 to 5 h of growth (Figure 4), showing a fluidization of the plasma membrane along germination. For wild type cells, STS did not have any significant effect on this parameter, suggesting that the effects sensed by *t*-PnA fluorescence intensity decays are localized into specific membrane domains, since the global properties of the membrane remain essentially unchanged.

Concerning the abc3 mutant, for 3 h growth, DPH steady-state fluorescence anisotropy reports no statistically significant effect of STS. The steady-state fluorescence anisotropy of DPH for abc3 cells was ca. 0.24 at 3 h of growth independently of the presence of STS. With 6 h of growth, the anisotropy value of the control decreased to ~0.18, following the general trend already reported for the wild type, with the plasma membrane becoming more fluid along the germination process. However, for this growth time, upon STS challenge, the DPH anisotropy of abc3 undergoes a marked increase to ~0.21. For this growth time, abc3 cells present a trend quite different from the wild type cells, since the membrane of the mutant cells becomes much more rigid with the STS challenge, in fact recovering from a control situation of higher fluidity, attaining a membrane fluidity that is between the control for 3 and 6 h of growth. This increased rigidity of the membrane as a whole is a biophysical response observed only for the mutant that is unable to efficiently export the drug.

**Figure 5:**
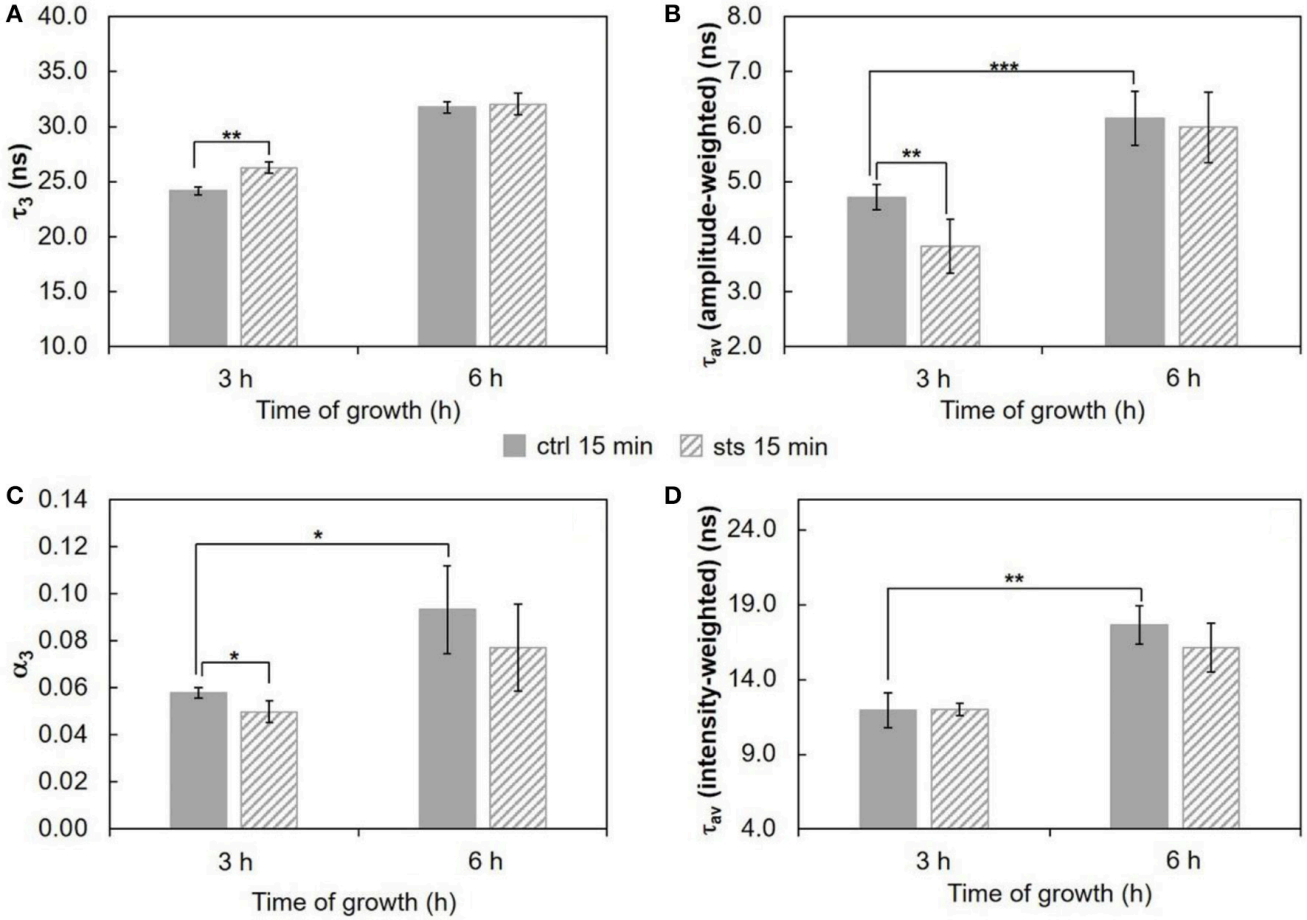
Time-resolved fluorescence spectroscopic parameters of *t*-PnA in the plasma membrane of *N. crassa* conidia. **(A)** The long lifetime component, τ_3_, **(B)** amplitude-weighted mean fluorescence lifetime, τ_av_ (amplitude-weighted), **(C)** normalized amplitude of the long component, α_3_, and **(D)** intensity-weighted mean fluorescence lifetime, τ_av_ (intensity-weighted), of *t*-PnA were obtained from the fluorescence intensity decay of the probe at 30°C. The results present the mean ± S.D. of at least three independent experiments, *n* ≥ 3. ^*^p < 0.05, ^**^p < 0.01, ^***^p < 0.001.

Wild type cells were also subjected to a 15 min challenge. In these conditions, the long lifetime component of *t*-PnA (Figure 5A and Table S3) showed only a change on the 3 h-grown cells, increasing ca. 8% from ~24 ns in the control to ~26 ns in the presence of STS, i.e., the drug is inducing tighter packing of the acyl chains in the more ordered domains. The amplitude (Figure 5C) associated with this long lifetime component presents the opposite behavior, decreasing with the addition of STS. This is also reflected in the amplitude-weighted mean fluorescence lifetime (Figure 5B) but not on the intensity-weighted mean fluorescence lifetime (Figure 5D). In the case of 6 h-grown cells, there were no significant changes, but in this case the long lifetime is already very high, even in the control situation. What is clear is that after 3 h growth a 15 min challenge with STS induces a rearrangement of sphingolipid-enriched domains. Considering the steady-state fluorescence anisotropy of DPH (not shown), no significant changes induced by STS could be perceived, as observed for the 1 h stimulus.

#### Polarity changes at the lipid/water interface

**Figure 6:**
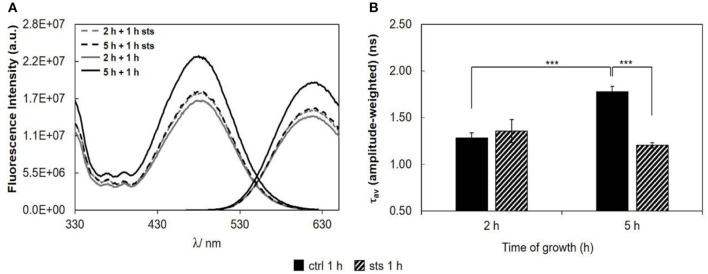
Di-4-ANEPPS fluorescence properties in *N. crassa* cells, at 30°C, **(A)** fluorescence excitation (λ_em_ = 635 nm) and emission spectra (λ_exc_ = 450 nm), **(B)** amplitude-weighted mean fluorescence lifetime of di-4-ANEPPS. The results present the mean ± S.D. of at least three independent experiments, *n* ≥ 3. ^***^p < 0.001.

To evaluate if STS can affect *N. crassa* plasma membrane polarity properties, conidia were labeled with di-4-ANEPPS, a probe from the class of potential sensitive naphtylstyryl dyes, extremely responsive to either ergosterol- or cholesterol-enriched lipid domains (Loew, 1996; Bastos et al., 2012a; Amaro et al., 2017). STS did not shift the emission or the excitation spectra of di-4-ANEPPS (Figure 6A, see also Figure S1, Tables S4–S6). However, the intensities of emission and excitation bands presented consistent variations that matched the trend of the amplitude-weighted mean fluorescence lifetime (Figure 6B). At 5 h growth, a significant decrease in the amplitude-weighted mean fluorescence lifetime with the addition of STS was observed. This decrease at 5 h of growth is contrary to the behavior of *t*-PnA fluorescence intensity decays (Figure 3), implying that di-4-ANEPPS is probably reporting the plasma membrane surface behavior of different domains than *t*-PnA.

**Figure 7:**
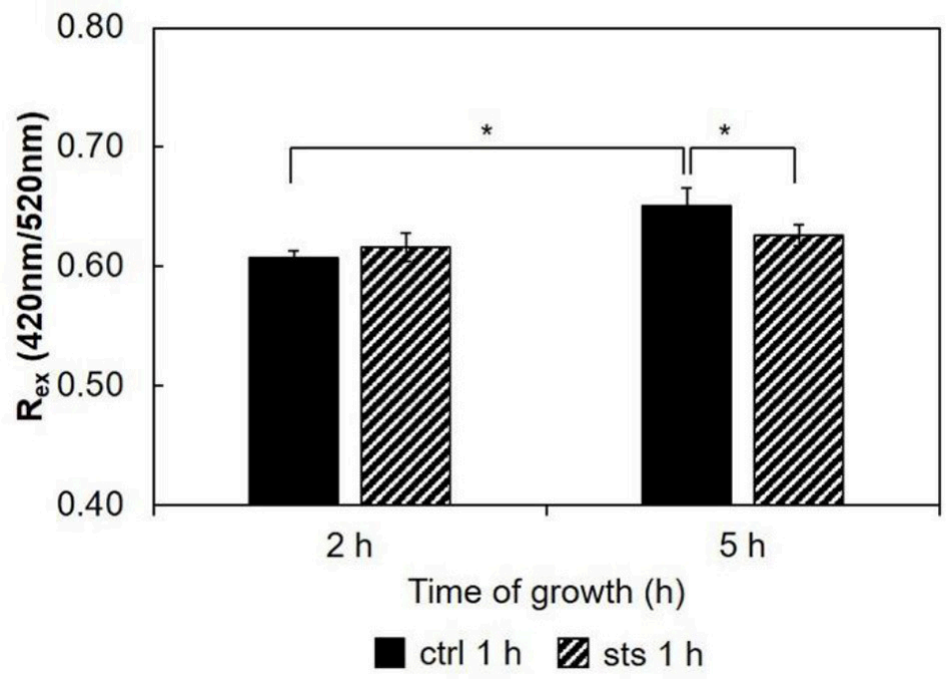
Ratio of di-4-ANEPPS fluorescence intensity produced by excitation at 420 nm to that produced by excitation at 520 nm, R_ex_ (420 nm/520 nm) cells, at 30°C. The results present the mean ± S.D. of at least three independent experiments, *n* ≥ 3. ^*^p < 0.05.

As a measure of the membrane dipole potential, the ratio of the di-4-ANEPPS fluorescence intensity by excitation at 420 nm to that produced by excitation at 520 nm (*R*_*ex*_ = IF_420_/IF_520_) was calculated (Figure 7). On 5 h-grown cells, STS significantly decreased *R*_*ex*_, from ~0.65 to ~0.61. This behavior corroborates the results of Figure 6, where both the steady-state fluorescence intensity and amplitude-weighted mean fluorescence lifetime of di-4-ANEPPS show similar trends. We can now suggest that these differences may be due to larger ergosterol content, since despite the absence of shifts of the spectra whether in the absence or in the presence of STS, this sterol is known to increase all the other photophysical parameters of di-4-ANEPPS showed in Figures 6, 7 (Bastos et al., 2012a).

**Figure 8:**
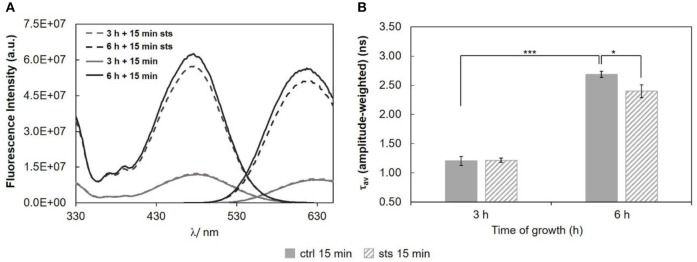
Di-4-ANEPPS fluorescence properties in *N. crassa* cells, at 30°C, **(A)** fluorescence excitation (λ_em_ = 635 nm) and emission spectra (λ_exc_ = 450 nm), **(B)** amplitude-weighted mean fluorescence lifetime of di-4-ANEPPS. The results present the mean ± S.D. of at least three independent experiments, *n* ≥ 3. ^*^p < 0.05, ^***^p < 0.001.

Regarding the 15 min stimulus (Figure 8), the effect of STS on the steady-state fluorescence intensity and amplitude-weighted mean fluorescence lifetime of di-4-ANEPPS is similar to the one obtained for 1 h stimulus (Figure 6), however, less pronounced at both growth times.

**Figure 9:**
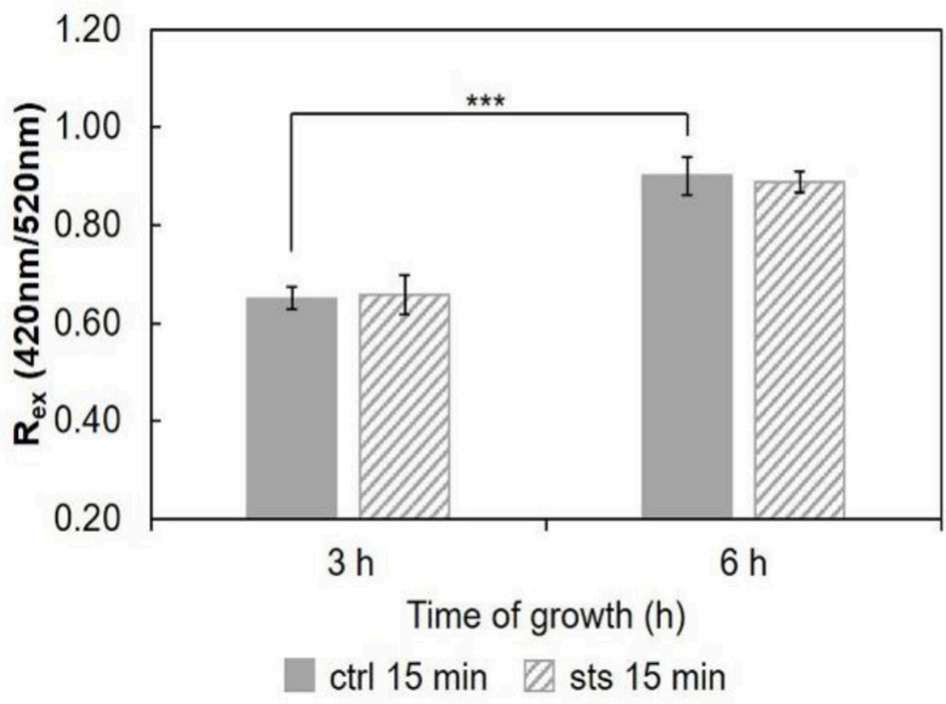
Ratio of di-4-ANEPPS fluorescence intensity produced by excitation at 420 nm to that produced by excitation at 520 nm, R_ex_ (420 nm/520 nm) in cells, at 30°C. The results present the mean ± S.D. of at least three independent experiments, *n* ≥ 3. ^***^p < 0.001.

Considering the ratio, *R*_*ex*_ = IF_420_/IF_520_, for the 15 min STS challenge (Figure 9), there were no significant changes induced by STS at both growth times. Nonetheless, note that the ratio at 6 h growth is ca. 0.9. Regarding the control, this value is considerably higher than that observed for any of the shorter growth times (Figure 7 and Santos et al., 2017), again reflecting the change in membrane composition. This abrupt change coincides with the transition from the conidial to mycelial stage, where a marked increase of ergosterol content occurs.

STS does not interact directly with membrane lipids (Section STS-Lipid Interactions). Thus, the differences observed in the membrane dipole potential, namely the increase with growth time and the decrease at 5 h growth upon incubation with STS relative to the control, can be also explained by the sterol content (Renaud et al., 1978). The 1 h treatment with the drug probably hampers the development of the fungus, and the change of growth phase from latency (*lag*) to exponential (*log*), when mycelium forms, preventing the rise in ergosterol content and membrane dipole potential.

## Discussion

Previous studies in *N. crassa* conidia showed that it is possible to establish relationships between lipid composition and dynamics during conidial growth (Santos et al., 2017). In this work, this relationship was further exploited by studying the influence of an STS challenge. The effects of STS were particularly noticeable for the 1 h treatment of conidia germinated for 5 h. These are the conditions for which ABC-3 protein reaches its maximum levels at the plasma membrane. At that time point, the process of STS-driven programmed cell death is at its early stage: reactive oxygen species had formed, glutathione efflux is at its maximum, but the integrity of the membrane is not yet affected (Fernandes et al., 2013).

The intracellular accumulation of STS over time was thoroughly studied (Fernandes et al., 2011). There is a higher accumulation of STS at 15 min than at 1 h probably due to STS export through ABC-3. However, we observe much stronger changes in membrane properties (related to ergosterol content) reported by di-4-ANEPPS for the 1 h treatment than for the 15 min, interestingly, when we confront the results of *t*-PnA fluorescence intensity decay for the two STS challenge times, it is possible to observe an opposite behavior for most of the changing parameters. Altogether, these results suggest a different response for the shorter and longer STS treatments. Upon 15 min treatment with STS the significant changes reported by *t*-PnA are observed only in 3 h-grown cells. There is an increase in the acyl chain packing of the more ordered domains, possibly associated with the increment of the levels of sphingolipids with smaller headgroups (e.g., ceramides, which are known to increase in certain apoptotic routes), which induce tighter packing of the lipid acyl chains (Lester et al., 1974). Although the increase in the value of τ_3_ seems modest (ca. 2 ns), and the significance is *p* < 0.05, once this value is converted to the rate constant for non-radiative processes, *k*_nr_, concerning the long-lived excited-state *t*-PnA molecules, these values become (2.86 ± 0.08) × 10^7^ s^−1^ and (3.19 ± 0.06) × 10^7^ s^−1^, respectively for the control and treated cells, i.e., the effect surpasses 10%, and the statistical significance is *p* < 0.01). The constant *k*_nr_ is directly related to the molecular motions and collisions responsible for the non-emissive relaxation of the excited probe molecules, and thus to the motions of the surrounding lipid acyl chains, reflecting the acyl chain packing of the lipids under physiological conditions, provided that probe molecules are highly diluted in the membrane, which is the case under our experimental conditions. This stronger segregation of sphingolipids contributes to a decrease in their abundance (α_3_). Both these alterations suggest an increased sphingolipid catabolism, which might be associated with the initiation of pro-apoptotic signaling. For the 1 h STS challenge, an increase in the sphingolipid-enriched domain abundance (α_3_) is observed instead for both growth times, without any change in the acyl chain packing.

On another hand, there were no changes in the photophysical properties of di-4-ANEPPS at the early conidial stage (shorter growth times), in clear contrast to what happens for longer growth times, at which the fungus has larger ability to express the STS exporter ABC-3. Being a very large transmembrane protein, from a major family that has been associated to lipid metabolism and transport (Li and Prinz, 2004), and to sphingolipid-enriched domains and/ or lipid rafts (Hinrichs et al., 2004; Modok et al., 2004), it is conceivable that ABC-3 expression induces changes on the membrane organization, especially in ordered domains, i.e., those reported, on one hand, by *t*-PnA (abundance increase) and on the other hand by di-4-ANEPPS, which abundance seems to be decreasing, since the increase of ergosterol levels is being limited by the treatment with STS. STS does not induce a marked expression of ABC-3 in 2 h-grown *N. crassa* conidia. The hypothetical involvement of membrane domains containing ABC-3 in the observed biophysical changes is therefore consistent with the fact that these changes are minor in 2 h-grown cells and upon short STS incubations when compared with the 5 h-grown cells, along with 1 h STS incubation.

Genes related to ion channel activity and ion pumps (Na^+^-K^+^ ATPase) are overexpressed in *N. crassa* conidia upon STS stimulus (Nagata et al., 2008; Fernandes et al., 2011). Thus, membrane dipole potential variations (Table 1), which are known to be intimately related with sterol composition and levels, might also be due to changes in lipid rafts that modulate ion channel activity (Ostroumova et al., 2015).

**Table 1:** Values of dipole potential ψ_d_ in mV obtained through Equation 1.

**Growth time**	**2 h**	**5 h**	**3 h**	**6 h**
Treatment time	1 h		15 min	
Control	211 ± 1	221 ± 4	221 ± 5	279 ± 9
STS treatment	213 ± 3	212 ± 1	223 ± 9	277 ± 5

*The values represent the mean ± S.D. of at least three independent experiments, n ≥ 3*.

It is known that, depending on the membrane composition, the magnitude of the dipole potential can range from 200 to 400 mV (Ostroumova et al., 2015). So far, it is possible to affirm that the *N. crassa* membrane dipole potential is within that range. The dipole potential of DPPC bilayers (one of the model systems used in this work) is 243 ± 4 mV (Peterson et al., 2002), and the dipole potential of neutral dioleoylphosphatidylethanolamine (DOPE) membranes is 220 ± 5 mV (Pickar and Benz, 1978; Cseh and Benz, 1998). The known lipid composition of *N. crassa* in the conidial and in the mycelium stages (Bianchi and Turian, 1967; Lester et al., 1974; Kushwaha et al., 1976; Renaud et al., 1978) suggest that, during culture growth and development, the ratio phosphatidylethanolamine (PE)/ phosphatidylcholine (PC) increases, which might also contribute to the increase in dipole potential, in addition to the aforementioned ergosterol.

**Figure 10:**
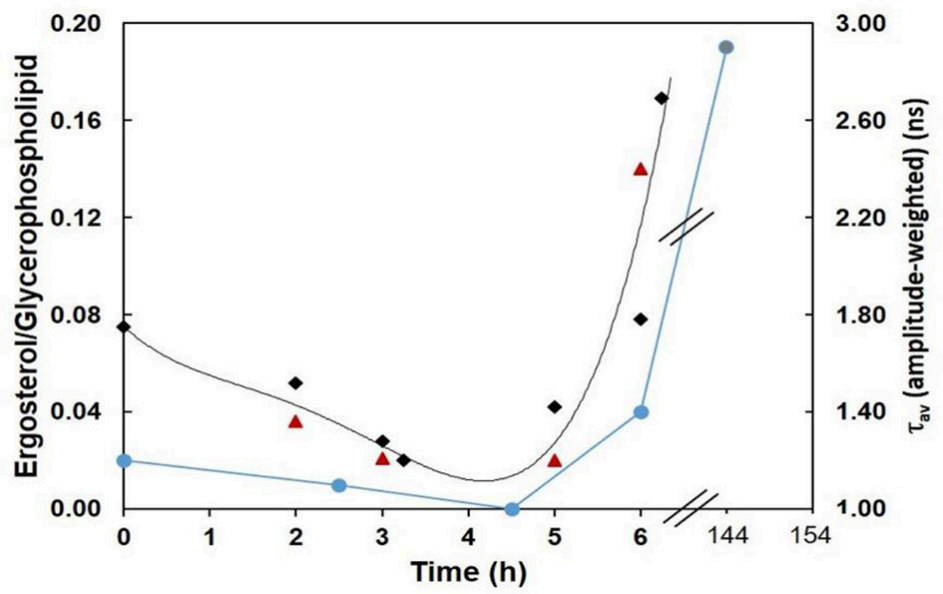
Ergosterol/Glycerophospholipid ratio (Bianchi and Turian, 1967; Kushwaha et al., 1976) (blue), and amplitude-weighted mean fluorescence lifetime of di-4-ANEPPS at 30°C, in the absence (CTRL, black) or in the presence of STS (red) vs. time of growth (this work and Santos et al., 2017). The last blue data point corresponds to the composition of mycelium (6 days growth). The lines are merely to guide the eye.

To evaluate to what extent the amplitude-weighted mean fluorescence lifetime of di-4-ANEPPS is indeed related to ergosterol levels, measures of that parameter (in the absence of STS) from this and previous work (Santos et al., 2017) were plotted against total time of growth (black points) and compared with the ergosterol/glycerophospholipid ratios (blue points) (Bianchi and Turian, 1967; Kushwaha et al., 1976) (Figure 10). The overall trend of di-4-ANEPPS τ_av_ correlates remarkably with what happens for ergosterol/glycerophospholipid ratios, both of them departing from a small value that further decreases, to increase exponentially upon starting of the *exponential* phase. In this figure it can also be more easily observed how STS prevents the marked increase observed upon transition to the *log* phase of di-4-ANEPPS τ_av_, The amplitude-weighted mean fluorescence lifetime of di-4-ANEPPS for STS treated cells (red triangles) is represented considering that growth stopped upon the addition of the drug. These points have a similar trend as the controls (black points) and are reasonably well described by the polynomial curve obtained from the fit to the controls. These effects can thus be putatively thought of as the result of a growth arrest or a slow-down in the development of the fungus. Despite the induction of a number of classical apoptotic markers, including caspase-like activity and surface binding of annexin V, in other organisms, such as *Leishmania donovani*, STS does not cause cell death but causes cell cycle arrest (Foucher et al., 2013). This arrest can be a direct consequence of the signaling pathways induced by STS, but might also be due to the targeting of energetic and metabolic resources for the synthesis of ABC-3 and other proteins involved in drug resistance mechanisms.

For the membrane dipole potential the effects of 1 h STS treatment and 5 h growth (the only case where they are significant) also correspond to a reversal of changes that occur between 5 h (+1 h) and shorter culture growth times. In the 6 h-grown abc3 mutant, the plasma membrane global fluidity is lower than for the wild type (control). In the presence of the drug, the intracellular level of STS remains high in abc3 cells even after 1 h of addition of the drug, and higher than for the wild type at 15 min (Fernandes et al., 2011). However, there is a strong decrease in global membrane fluidity (increased DPH anisotropy) of the abc3 mutant membrane upon STS 1 h challenge, of the membrane exposed to the drug as a whole, attaining a value that is intermediate between its control values for 2 h and 5 h growth.

## Conclusions

This work clearly demonstrates that filamentous fungi response to STS involves changes of plasma membrane biophysical properties even though the drug does not interact directly with membrane lipids. Sphingolipid-enriched domains and sphingolipid metabolism and/or traffic to the plasma membrane seem to be involved in both fast and slow responses to the drug, by different mechanisms. Furthermore, ergosterol and possibly ergosterol-enriched domains may be crucial for the transition to the exponential phase and mycelium formation. Investigation on the relationships between drug export and sterol levels/enriched domains and membrane biophysical properties is worth pursuing in the context of antifungal resistance and eventually other drug resistance situations, such as drug resistant cancer cell lines expressing proteins of the ABC-3 family, like the P-glycoprotein.

## Author contributions

FS, GL, and AF: performed research and analyzed data; AF and AV: edited the manuscript; AV, RdA, and AF: designed the project; FS and RdA: designed research and wrote the manuscript; RdA: supervised and coordinated the research.

### Conflict of interest statement

The authors declare that the research was conducted in the absence of any commercial or financial relationships that could be construed as a potential conflict of interest.

## Abbreviations

ABC: ATP Binding Cassette
Di-4-ANEPPS: 4-(2-(6-(dibutylamino)-2-naphthalenyl)ethenyl)-1-(3-sulfopropyl)-pyridinium
DOPC: 1,2-dipalmitoyl-sn-glycero-3-phosphocholine
DPH: 1,6-diphenyl-1,3,5-hexatriene
FGSC: Fungal Genetics Stock Center
POPC: 1-palmitoyl-2-oleoyl-sn-glycero-3-phosphocholine
STS: staurosporine
Tm: main transition temperature
t-PnA: trans-parinaric acid.
