# Supplementary material for: Changes in the Biophysical Properties of the Cell Membrane Are Involved in the Response of Neurospora crassa to Staurosporine
Source: Front Physiol. 2018 Oct 11;9:1375. doi: 10.3389/fphys.2018.01375 (PMC6193110; doi:10.3389/fphys.2018.01375)
Supplement: Supplementary file 1 [file Data_Sheet_1.pdf]

Supplementary Material

Changes in the biophysical properties of the cell membrane are involved in the response of *Neurospora crassa* to staurosporine

Filipa C. Santos[1], Gerson M. Lobo[1], Andreia S. Fernandes[2,3], Arnaldo Videira[2,3,4], Rodrigo F. M. de Almeida[1*]

[1] Centro de Química e Bioquímica, Departamento de Química e Bioquímica, Faculdade de Ciências, Universidade de Lisboa, Campo Grande, 1749-016 Lisboa, Portugal

[2] I3S - Instituto de Investigação e Inovação em Saúde, Universidade do Porto, Rua Alfredo Allen 208, 4200-135 Porto, Portugal

[3] IBMC-Instituto de Biologia Molecular e Celular, Universidade do Porto, Rua do Campo Alegre 823, 4150-180 Porto, Portugal

[4] ICBAS-Instituto de Ciências Biomédicas de Abel Salazar, Universidade do Porto, Rua de Jorge Viterbo Ferreira 228, 4050-313 Porto, Portugal

***Corresponding author:** Rodrigo F. M. de Almeida

Address: Centro de Química e Bioquímica, Faculdade de Ciências da Universidade de Lisboa, Edifício C8, Campo Grande, 1749-016 Lisboa, Portugal

e-mail: rodrigo.almeida@fc.ul.pt

Tel: +351 217 500 925 **Fax:** +351 217 500 088

Table S1 Selected data from the transcriptional analysis of the 1h STS challenge of *N. crassa* conidial cells grown for 5 h, taken from Fernandes, A.S., Goncalves, A.P., Castro, A., Lopes, T.A., Gardner, R., Glass, N.L., and Videira, A. (2011) *Fungal Genet Biol 48*, 1130-1138.

gene	sample_1	sample_2	status	value_1	value_2	log2(fold_change)	test_stat	p_value	q_value	significant	BROAD_Annotation_March2011	
NCU09975	wtDMSO	wtSTS	OK	6.78603	797.821	6.87735	-16.5023	0	0	yes	multidrug resistance protein 3	
NCU09473	wtDMSO	wtSTS	OK	20.6851	290.81	3.81341	-8.97906	0	0	yes	3-ketoacyl-acyl carrier protein reductase	3-oxo-acyl-[acyl-carrier-protein] reductase exhibits a marked preference for acyl-carrier protein derivatives over CoA derivatives as substrates. The enzyme shows activity towards both short and long chain saturated and unsaturated ß-ketoacyl-ACPs. The reductase functions in every cycle of the fatty acid elongation pathway. Other reductases of this type also exist in E. coli [Magnuson93, Heath96b]. The enzyme is also involved in the elongation of 3-ketoglutaryl-[acp]-methyl-ester to pimeloyl-[acp]-methyl-ester, part of the biotin biosynthesis pathway [Lin10].
NCU03929	wtDMSO	wtSTS	OK	18.793000	170.309000	3.179890	-8.584620	0.000000	0.000000	yes	acyl-CoA synthetase	
NCU08147	wtDMSO	wtSTS	OK	7.856650	108.687000	3.790120	-9.747290	0.000000	0.000000	yes	Na or K P-type ATPase	
NCU03639	wtDMSO	wtSTS	OK	29.851900	266.787000	3.159790	-8.160950	0.000000	0.000000	yes	lipase	
NCU05899	wtDMSO	wtSTS	OK	4.904470	60.893400	3.634120	-8.134920	0.000000	0.000000	yes	flotillin domain-containing protein	
NCU09337	wtDMSO	wtSTS	OK	52.049000	4.835380	-3.428170	7.641030	0.000000	0.000000	yes	plasma membrane fusion protein prm-1	
NCU06005	wtDMSO	wtSTS	OK	46.748800	4.212840	-3.472060	7.543640	0.000000	0.000000	yes	glycerol kinase	
NCU02287	wtDMSO	wtSTS	OK	29.259000	176.423000	2.592080	-6.861070	0.000000	0.000000	yes	acyl-CoA dehydrogenase	
NCU00130	wtDMSO	wtSTS	OK	2.403480	25.253500	3.393290	-6.615720	0.000000	0.000000	yes	beta-glucosidase	
NCU01068	wtDMSO	wtSTS	OK	117.984000	21.670000	-2.444820	6.443370	0.000000	0.000000	yes	BAR domain-containing protein	
NCU04479	wtDMSO	wtSTS	OK	42.493000	223.192000	2.392990	-6.189490	0.000000	0.000000	yes	LAP2	
NCU08924	wtDMSO	wtSTS	OK	39.233900	184.905000	2.236620	-6.158780	0.000000	0.000000	yes	acyl-CoA dehydrogenase	
NCU02544	wtDMSO	wtSTS	OK	8.622140	47.559900	2.463630	-5.951730	0.000000	0.000000	yes	ABC transporter	
NCU07546	wtDMSO	wtSTS	OK	6.508890	33.511500	2.364170	-5.899320	0.000000	0.000000	yes	multidrug resistance protein MDR	
NCU11109	wtDMSO	wtSTS	OK	71.734200	299.789000	2.063210	-5.818880	0.000000	0.000000	yes	ORM1	Component of the SPOTS complex that acts as a negative regulator of sphingolipid synthesis. Acts by inhibiting serine palmitoyltransferases (LCB1 and LCB2) activity
NCU04021	wtDMSO	wtSTS	OK	4.925710	25.286800	2.359980	-5.794680	0.000000	0.000000	yes	ABC transporter	
NCU01107	wtDMSO	wtSTS	OK	5.716320	43.154000	2.916340	-5.626310	0.000000	0.000000	yes	short-chain dehydrogenase	
NCU04736	wtDMSO	wtSTS	OK	30.484000	117.254000	1.943520	-5.535560	0.000000	0.000001	yes	plasma membrane calcium-transporting ATPase 3	
NCU06694	wtDMSO	wtSTS	OK	138.923000	528.243000	1.926920	-5.488300	0.000000	0.000001	yes	fatty acid elongase	
NCU03768	wtDMSO	wtSTS	OK	49.993500	9.591930	-2.381850	5.236550	0.000000	0.000002	yes	lysophospholipase, variant 2	
NCU08372	wtDMSO	wtSTS	OK	0.519639	4.924010	3.244250	-5.162900	0.000000	0.000003	yes	triacylglycerol lipase	
NCU06364	wtDMSO	wtSTS	OK	23.155600	3.728430	-2.634720	5.097590	0.000000	0.000005	yes	GDSL lipase/acylhydrolase	
NCU08977	wtDMSO	wtSTS	OK	31.567300	117.428000	1.895280	-5.041450	0.000000	0.000006	yes	long chain fatty alcohol oxidase	
NCU05207	wtDMSO	wtSTS	OK	2.058190	14.680200	2.834420	-5.039550	0.000000	0.000006	yes	MFS multidrug transporter	
NCU12074	wtDMSO	wtSTS	OK	5.329910	28.140000	2.400440	-5.001480	0.000001	0.000007	yes	5-nitroimidazole antibiotic resistance protein	
NCU06382	wtDMSO	wtSTS	OK	5.399280	0.894413	-2.593750	4.986180	0.000001	0.000008	yes	ABC transporter	
NCU03497	wtDMSO	wtSTS	OK	22.075800	3.989470	-2.468190	4.916460	0.000001	0.000011	yes	plasma membrane iron permease	
NCU05627	wtDMSO	wtSTS	OK	41.486200	142.091000	1.776110	-4.850080	0.000001	0.000015	yes	high affinity glucose transporter ght1	
NCU02579	wtDMSO	wtSTS	OK	1.884410	12.033600	2.674890	-4.671830	0.000003	0.000033	yes	FAS1 domain-containing protein	
NCU01378	wtDMSO	wtSTS	OK	7.163080	34.178300	2.254430	-4.597220	0.000004	0.000046	yes	acetoacetyl-CoA synthase	
NCU09692	wtDMSO	wtSTS	OK	9.785690	1.477070	-2.727930	4.563700	0.000005	0.000053	yes	phosphatidic acid phosphatase beta	
NCU02726	wtDMSO	wtSTS	OK	52.019100	152.866000	1.555160	-4.236950	0.000023	0.000200	yes	ethanolamine kinase	
NCU01654	wtDMSO	wtSTS	OK	53.617000	133.763000	1.318910	-3.742520	0.000182	0.001255	yes	long-chain-fatty-acid-CoA ligase 1	
NCU09497	wtDMSO	wtSTS	OK	544.225000	216.566000	-1.329400	3.740650	0.000184	0.001264	yes	bifunctional D12/D15 fatty acid desaturase	
NCU08045	wtDMSO	wtSTS	OK	117.551000	312.121000	1.408820	-3.659310	0.000253	0.001672	yes	phosphatidylethanolamine N-methyltransferase	
NCU01004	wtDMSO	wtSTS	OK	31.210300	75.905100	1.282180	-3.612380	0.000303	0.001957	yes	phosphatidylserine decarboxylase proenzyme	
NCU03372	wtDMSO	wtSTS	OK	3.485350	11.710900	1.748470	-3.497490	0.000470	0.002856	yes	nonspecific lipid-transfer protein	
NCU06874	wtDMSO	wtSTS	OK	4.772800	14.085100	1.561260	-3.423350	0.000619	0.003618	yes	HMG box-containing protein	
NCU08976	wtDMSO	wtSTS	OK	344.536000	807.528000	1.228860	-3.419700	0.000627	0.003660	yes	fatty acid elongase	
NCU01112	wtDMSO	wtSTS	OK	0.238675	3.270530	3.776400	-3.388280	0.000703	0.004039	yes	lipid binding protein	
NCU04699	wtDMSO	wtSTS	OK	450.142000	1031.780000	1.196690	-3.373530	0.000742	0.004230	yes	methylene-fatty-acyl-phospholipid synthase	
NCU04092	wtDMSO	wtSTS	OK	13.474900	33.867600	1.329630	-3.278820	0.001042	0.005642	yes	N-acylethanolamine amidohydrolase	
NCU02175	wtDMSO	wtSTS	OK	5.431200	14.964700	1.462220	-2.977490	0.002906	0.013370	yes	phosphatidyl inositol-specific phospholipase C	
NCU06761	wtDMSO	wtSTS	OK	41.666000	87.120900	1.064150	-2.948890	0.003189	0.014443	yes	sphingosine-1-phosphate lyase	
NCU07307	wtDMSO	wtSTS	OK	176.841000	380.261000	1.104540	-2.942410	0.003257	0.014714	yes	fatty acid synthase beta subunit dehydratase	
NCU07308	wtDMSO	wtSTS	OK	195.555000	407.017000	1.057520	-2.833550	0.004603	0.019585	yes	fatty acid synthase alpha subunit reductase	
NCU01069	wtDMSO	wtSTS	OK	35.838900	73.054400	1.027440	-2.786030	0.005336	0.022090	yes	amphiphysin-like lipid raft protein	
NCU05278	wtDMSO	wtSTS	OK	111.279000	217.775000	0.968661	-2.748650	0.005984	0.024305	yes	cytochrome P450 61	(involved in C-22 desaturation of the ergosterol side-chain)
NCU05608	wtDMSO	wtSTS	OK	10.652800	21.556500	1.016890	-2.659850	0.007818	0.030214	yes	phosphatidylinositol 3-kinase tor2	

Table S2. Fluorescence Spectroscopy Parameters of *t*-PnA in *N. crassa* at 30 ºC, 2 h and 5 h growth, in the absence and presence of 1 h challenge with staurosporine.

Time of growth (h)	Incubation time (min; h)	α_1	τ_1 (ns)	α_2	τ_2 (ns)	α_3	τ_3 (ns)	τ_{av} (ns)	$<\tau>$ (ns)
2 h	1 h sts	0.64 ± 0.03	1.75 ± 0.09	0.30 ± 0.01	5.30 ± 0.19	0.06 ± 0.01	20.71 ± 1.11	3.88 ± 0.34	8.83 ± 1.15
	1 h ctrl	0.63 ± 0.01	1.52 ± 0.07	0.327 ± 0.008	4.91 ± 0.20	0.042 ± 0.003	20.44 ± 1.43	3.41 ± 0.03	7.81 ± 0.34
5 h	1 h sts	0.585 ± 0.004	2.03 ± 0.07	0.354 ± 0.005	5.90 ± 0.19	0.061 ± 0.008	24.51 ± 0.38	4.78 ± 0.27	10.78 ± 0.66
	1 h ctrl	0.64 ± 0.03	2.11 ± 0.09	0.31± 0.03	5.91 ± 0.28	0.047 ± 0.002	24.11 ± 0.59	4.31 ± 0.03	9.50 ± 0.10

Table S3. Fluorescence Spectroscopy Parameters of *t*-PnA in *N. crassa* at 30 ºC, 3 h and 6 h growth, in the absence and presence of 15 min challenge with staurosporine.

Time of growth (h)	Incubation time (min; h)	α_1	τ_1 (ns)	α_2	τ_2 (ns)	α_3	τ_3 (ns)	τ_{av} (ns)	<τ> (ns)
3 h	15 min sts	0.63 ± 0.04	1.73 ± 0.31	0.31 ± 0.04	6.13 ± 0.44	0.050 ± 0.005	26.27 ± 0.52	3.83 ± 0.49	11.98 ± 0.41
	15 min ctrl	0.581 ± 0.004	1.38 ± 0.07	0.356 ± 0.006	6.44 ± 0.25	0.058 ± 0.002	24.17 ± 0.36	4.71 ± 0.23	11.94 ± 1.18
6 h	15 min sts	0.474 ± 0.002	1.86 ± 0.06	0.44 ± 0.02	6.34 ± 0.40	0.08 ± 0.02	32.04 ± 0.99	5.99 ± 0.64	16.13 ± 1.65
	15 min ctrl	0.48 ± 0.06	1.59 ± 0.20	0.43 ± 0.04	5.71 ± 0.34	0.09 ± 0.02	31.77 ± 0.53	6.15 ± 0.49	17.64 ± 1.28

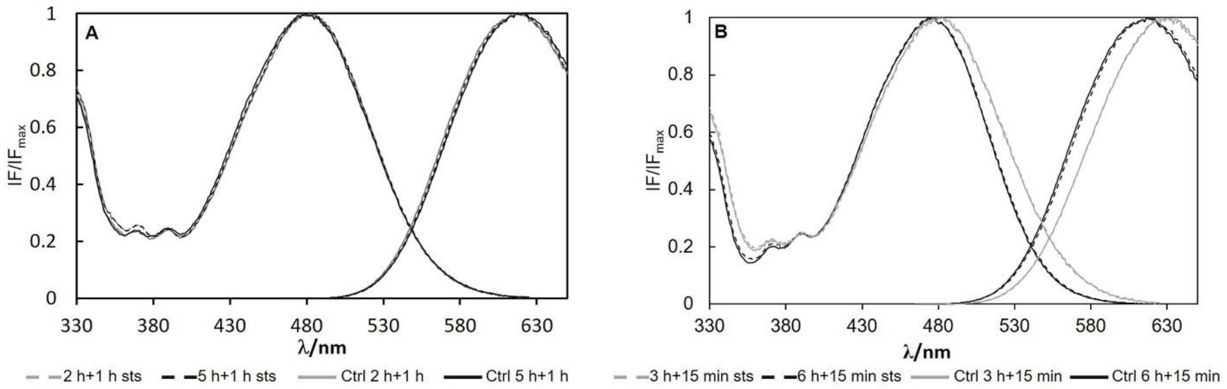

Figure S1. Fluorescence excitation (λ_{em} = 635 nm) and emission spectra (λ_{exc} = 450 nm) of di-4-ANEPPS in *N. crassa* WT cells, at 30 ºC. **(A)** at 2 and 5 h growth plus 1 h incubation with STS, **(B)** at 3 and 6 h growth plus 15 min incubation with STS. The represented spectra are the mean of at least three independent experiments, n ≥ 3.

Table S4. Maximal emission and excitation wavelengths for di-4-ANEPPS in *N. crassa* at 30 ºC, in the absence and presence of staurosporine.

Time of growth (h)	2 h		5 h		3 h		6 h	
**Incubation time (min;h)**	**1 h sts**	**1 h ctrl**	**1 h sts**	**1 h ctrl**	**15 min sts**	**15 min ctrl**	**15 min sts**	**15 min ctrl**
**$\lambda_{em\ max}$ (nm)**	619 ± 1	619 ± 1	618 ± 1	617 ± 1	624 ± 3	627 ± 2	617 ± 3	617 ± 1
**$\lambda_{exc\ max}$ (nm)**	483 ± 1	484 ± 2	483 ± 2	480 ± 1	480 ± 3	483 ± 1	477 ± 2	476 ± 1

Table S5. Fluorescence Spectroscopy Parameters of di-4-ANEPPS in *N. crassa* at 30 ºC, 2 h and 5 h growth, in the absence and presence of 1 h challenge with staurosporine.

**Time of growth (h)**	**Incubation time (min; h)**	α_1	τ_1 **(ns)**	α_2	τ_2 **(ns)**	α_3	τ_3 **(ns)**	τ_{av} **(ns)**	$\langle\tau\rangle$ **(ns)**
2 h	1 h sts	0.44 ± 0.05	0.25 ± 0.02	0.35 ± 0.02	1.49 ± 0.01	0.21 ± 0.03	3.46 ± 0.01	1.36 ± 0.12	2.44 ± 0.07
	1 h ctrl	0.48 ± 0.02	0.210 ± 0.009	0.32 ± 0.01	1.48 ± 0.07	0.21 ± 0.01	3.48 ± 0.06	1.28 ± 0.06	2.49 ± 0.08
5 h	1 h sts	0.51 ± 0.02	0.21 ± 0.02	0.31 ± 0.01	1.50 ± 0.08	0.18 ± 0.02	3.52 ± 0.06	1.20 ± 0.03	2.43 ± 0.03
	1 h ctrl	0.34 ± 0.04	0.50 ± 0.01	0.42 ± 0.03	1.736 ± 0.003	0.242 ± 0.005	3.63 ± 0.04	1.78 ± 0.06	2.56 ± 0.03

Table S6. Fluorescence Spectroscopy Parameters of di-4-ANEPPS in *N. crassa* at 30 ºC, 3 h and 6 h growth, in the absence and presence of 15 min challenge with staurosporine.

**Time of growth (h)**	**Incubation time (min; h)**	α_1	τ_1 **(ns)**	α_2	τ_2 **(ns)**	α_3	τ_3 **(ns)**	τ_{av} **(ns)**	$\langle\tau\rangle$ **(ns)**
3 h	15 min sts	0.52 ± 0.02	0.37 ± 0.02	0.349 ± 0.008	1.63 ± 0.07	0.14 ± 0.02	3.38 ± 0.12	1.22 ± 0.04	2.08 ± 0.02
	15 min ctrl	0.49 ± 0.03	0.34 ± 0.08	0.348 ± 0.008	1.46 ± 0.12	0.17 ± 0.03	3.18 ± 0.09	1.20 ± 0.08	2.07 ± 0.04
6 h	15 min sts	0.18 ± 0.02	0.458 ± 0.006	0.46 ± 0.04	2.19 ± 0.01	0.36 ± 0.06	3.67 ± 0.05	2.40 ± 0.11	2.93 ± 0.08
	15 min ctrl	0.15 ± 0.08	0.94 ± 0.06	0.48 ± 0.06	2.51 ± 0.33	0.97 ± 0.10	3.64 ± 0.08	2.69 ± 0.05	3.01 ± 0.02
